# Evaluation of the total volatile basic nitrogen (TVB-N) content in fish fillets using hyperspectral imaging coupled with deep learning neural network and meta-analysis

**DOI:** 10.1038/s41598-021-84659-y

**Published:** 2021-03-03

**Authors:** Marzieh Moosavi-Nasab, Sara Khoshnoudi-Nia, Zohreh Azimifar, Shima Kamyab

**Affiliations:** 1 Seafood Processing Research Group, Department of Food Science and Technology, School of Agriculture, Shiraz University, P.O. Box 71441-65186, Shiraz, Iran; 2 Seafood Processing Research Group, School of Agriculture, Shiraz University, P.O. Box 71441-65186, Shiraz, Iran; 3 Department of Computer Science and Engineering, Shiraz University, P.O. Box 71936-16548, Shiraz, Iran

**Keywords:** Analytical chemistry, Computational models

## Abstract

Recently, hyperspectral-imaging (HSI), as a rapid and non-destructive technique, has generated much interest due to its unique potential to monitor food quality and safety. The specific aim of the study is to investigate the potential of the HSI (430–1010 nm) coupled with Linear Deep Neural Network (LDNN) to predict the TVB-N content of rainbow trout fillet during 12 days storage at 4 ± 2 °C. After the acquisition of hyperspectral images, the TVB-N content of fish fillets was obtained by a conventional method (micro-Kjeldahl distillation). To simplify the calibration models, nine optimal wavelengths were selected by the successive projections algorithm. A seven layers LDNN was designed to estimate the TVB-N content of samples. The LDNN model showed acceptable performance for prediction of TVB-N content of fish fillet (R^2^p = 0.853; RSMEP = 3.159 and RDP = 3.001). The performance of LDNN model was comparable with the results of previous works. Although, the results of the meta-analysis did not show any significant difference between various chemometric models. However, the least-squares support vector machine algorithm showed better prediction results as compared to the other models (RMSEP: 2.63 and R^2^_p_ = 0.897). Further studies are required to improve the prediction power of the deep learning model for prediction of rainbow-trout fish quality.

## Introduction

Recently, hyperspectral imaging (HSI), as a rapid and non-invasive measurement ability, has generated much interest due to its unique potential to monitor food quality and safety^1–3^. Among various food materials, fish is considered one of the most perishable foods. Consumption of deteriorated fish can seriously affect consumer health. Therefore, to evaluate and monitor the quality and safety of this valuable and at the same perishable seafood, the rapid and non-destructive detection of freshness is a necessary task^4,5^. Hyperspectral imaging method in combination with different chemometric analysis has been applied to evaluate several freshness indicators, such as Total Volatile Basic Nitrogen (TVB-N), Trimethylamine (TMA)^5–7^, Thiobarbituric acid reactive substances (TBARS)^8–10^, total viable count (TVC)^11–13^, sensory factors^5,14,15^ and, etc.

Volatile compounds such as trimethylamine, ammonia and dimethylamine are considered as total volatile basic nitrogen (TVB-N), produced as a result of destructive activities of microorganisms and are considered as one of the most important freshness indicators to monitor the quality and safety of seafood products^16^. During storage time, the change of the TVB-N value of fish fillets caused several chemical variations that can be shown in the hyperspectral imaging data and be applied to measure the TVB-N value.

Although the capability of HSI for evaluating the TVB-N value of fish fillet has already been proved, attempts to improve the prediction power of HSI is still considered as an attractive subject. One of the most important efforts to enhance the performance of hyperspectral imaging technique could be to focus on new chemometric methods. Many data analysis methods such as partial least squares (PLS), random forest, principal component analysis (PCA), support vector machine (SVM), artificial neural network (ANN) and so on have been developed to deal with the large volume of data.

Deep learning, as a powerful machine learning tool, is a promising method to be used in various fields such as medical science, remote sensing, robotic and food safety, etc. This technique dealing with huge data sets, and obtaining suitable prediction power and accuracy. Deep learning is a kind of representation-learning method that refines multilevel representation by the deep artificial neural network composed by multiple layers of neurons. Because of the strong feature learning capability, deep learning method can solve many complicated problems in a rapid and effective manner^17^. Deep learning models exhibit powerful ability in classification and regression tasks^18^. There are several studies, using deep learning techniques in the recent literature, which indicates the superiority of this class of machine learning techniques in the food industry by analyzing RGB images and spectra images of food. For example, Yu et al. (2017) used visible and near-infrared hyperspectral imaging (HSI) technique combined with stacked auto-encoders (SAEs) algorithm to classify shrimp into fresh and stale groups according to their TVB-N contents. This method achieved satisfactory total classification accuracy of 96.55 (for the calibration set with 116 samples) and 93.97% (for the prediction set with 116 samples) freshness grade of shrimp^19^. Al-Sarayreh et al. (2018) compared the performance of HSI data to evaluate adulteration in red-meat by the support vector machines and deep convolution neural networks (CNN) algorithm. Results confirmed that the CNN model has the best prediction power (94.4% classification accuracy)^20^. Moreover, Yu et al. (2019) used the hyperspectral imaging (HSI: 900–1700 nm) technique to predict the TVB-N value of Pacific white shrimp. They used Successive projections algorithm (SPA) and deep-learning-based stacked auto-encoders algorithms to choose more informative wavelengths. Least-squares support vector machine (LS-SVM), partial least squares regression (PLSR) and multiple linear regression (MLR) were applied to predict TVB-N content. The results demonstrated that the SAEs-LS-SVM was the best model (R_P_^2^ = 0.921, RMSEP = 6.22 mg N [100 g]^−1^ and RPD = 3.58)^21^ for prediction of this index. Overall, in these researches, deep learning was applied to classify the samples or select optimal wavebands. It is sometimes necessary to evaluate the numerical output of a product's freshness index for better decision making. Therefore, the use of a regression framework can be preferred over a classification one. To the best knowledge, no research has been reported yet on the detection of fish quality by a regression framework of deep learning. Moreover, systematization and meta-analysis of the data extracted from previous studies can help to obtain conclusive results about the best predictive model for evaluating the TVB-N content of fish fillets. Therefore, the discussion of results was established based on meta-analysis and systematic review. Therefore, the specific aim of the study is to (1) investigate the potential of visible and near-infrared (VIS/NIR) hyperspectral imaging technique coupled with deep learning model to predict the TVB-N content of rainbow trout fish, and (2) compare the performance of deep learning algorithm with PLSR and LS-SVM models established in current study and (3) meta-analysis of previous researches on the prediction of the TVB-N value in meat products using hyperspectral imaging coupled with various chemometric algorithms.

## Results and discussion

### TVB-N value

Changes in TVB-N values of 210 subsamples (30 fish fillets per day) during storage were presented in Table 1. The initial TVB-N content of the rainbow trout fillets was 8.70 ± 0.86 N/100 g, which significantly increased during storage time and finally reached to 36.79 ± 4.38 N/100 g, which this data is comparable with previous study results for rainbow trout fish fillets^22–25^.

**Table 1:** Descriptive statistics for TVB-N content of samples, measured by the conventional methods.

Variable	N	Mean ± StDev	SE	Variance	Minimum	Maximum
0th day	30	8.70 ± 0.86^G^	0.156	0.73	7.31	11.16
2nd day	30	11.53 ± 1.24^F^	0.226	1.54	9.58	15.51
4th day	30	14.39 ± 1.39^E^	0.253	1.92	11.28	17.11
6th day	30	17.14 ± 1.67^D^	0.305	2.79	14.21	20.66
8th day	30	20.49 ± 2.07^C^	0.379	4.31	16.28	25.49
10th day	30	26.78 ± 3.63^B^	0.663	13.19	21.16	35.16
12th day	30	36.79 ± 4.38^A^	0.799	19.16	28.71	46.11
Calibration (set)	140	19.52 ± 9.29	0.785	86.33	7.31	46.11
Prediction (set)	70	19.17 ± 9.48	1.13	89.95	7.65	43.13
All	210	19.40 ± 9.34	0.64	87.14	7.31	46.11

Different letters indicate significant difference between average of samples (*p* < 0.05).

The threshold limit of acceptability for the TVB-N of rainbow trout, as a freshwater fish is considered 20 N/100g^26–28^. Based on this critical value, the acceptable shelf-life for analyzed rainbow trout was 8 days. Furthermore, as shown in Table 1. the variation range of TVB-N for calibration and prediction set were 38.8 and 35.48 mg N/100 g, respectively. Therefore, the differences between the fresh and stale samples were highlighted during 12 days storage which was helpful to establish a suitable and robust calibration model for predicting the total volatile basic nitrogen and consequently estimating shelf-life and quality of rainbow trout fish subsamples^29^.

### Spectral feature analysis

**Figure 1:**
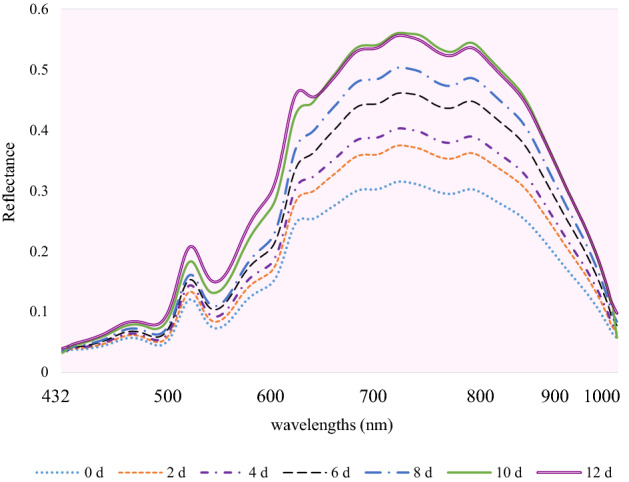
Extracted mean reflectance spectra of rainbow trout fish fillets during cold storage.

The mean reflectance spectra plot (400–1000 nm) of fish fillet with different TVB-N values are illustrated in Fig. 1. The spectral reflectance curves of samples in various storage days followed an almost similar trend. However, overtime, there was an increase in spectral reflectance across the whole investigated waveband range. The amplitude of variation of spectral reflectance was recognizable on the spectra plot of samples. it was mainly related to the chemical and physical variations in fish muscle during the freshness loss^30,31^ due to microbial and enzyme activity. Hence, the fresh samples with lower TVB-N content showed the curves with lower reflectance and vice versa.

Overall, the bands in the visible range, 400–700 nm can be connected to the change of fish color. The significant absorption regions were observed around near infrared range (700–1000 nm) may be related to overtones of several chemical bonds, such as N–H (760–840 nm: protein), C-H (930 nm: protein compound), O–H (690–720 nm and 970 nm: water and lipid oxidation compound)and S–H (930 nm: methylene) stretching^8,13,14,32–34^.

### Optimal wavelength selection

**Figure 2:**
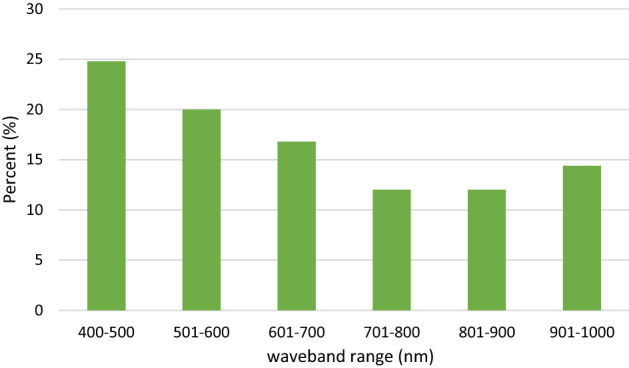
Frequency of optimal spectral range in previous studies.

In the current study, the SPA method was used to choose the most important wavebands related to the TVB-N content of fish quality from the full spectral range. Nine wavelengths (459, 552, 616, 629, 695, 760, 896, 956 and 986 nm) were considered as the optimal variable which covered the whole spectral range. Figure 2 showed the frequency of various waveband ranges selected by different methods in previous studies. As seen in Fig. 2 the optimal wavelengths almost covered the full spectral range. More than half of these wavebands (5 out of 9 wavebands) were located in the visible region of the spectrum (400–750 nm). The changes of the chemical compounds (e.g. protein, fat, water, etc.) occurring during freshness loss of fish can directly reflect in fish fillet color and result in the spectral variations in visible region^35^. These results were agreed with several previous studies (Fig. 2 and Table 3). Moreover, the results of the meta-analysis (Fig. 2) showed that the most frequent waveband range was located at 400–500 (25%) and 501–600 (20%) nm. However, in the current study, most of the selected wavebands were located at the main absorption range of 601–700 nm which is related to the variation of H_2_S produced by microbial activity^36^. The waveband of 890 nm is ascribed to the C–H and N–H stretching that is associated with protein, methylene group of lipid^11^. Water in the fish fillet is the major component and finally, the selected waveband 950 nm is assigned to the second overtone O–H stretching in water and the third overtone C–H and C–H_2_ stretching of fat^11,37^. The waveband was observed around 1000 nm (990 nm) which are mainly related to the NH stretching of proteins^11,21^.

### Evaluation of TVB-n value based on deep learning regression framework

In this work, hyperspectral imaging (HSI) coupled with linear deep neural network, as a chemometric algorithm, has been applied to evaluate the TVB-N content of rainbow-trout fish fillets. It is still very hard to obtain a big dataset for TVB-N value of fish samples, due to the use of manual, time consuming and destructive information acquisition tools. Therefore, the main characteristic of the available data is the small sample size (210 samples) which restricts the functionality of the machine learning tools which are used for prediction. Therefore, the use of the LDNN model was proposed for resolving this problem. Because by using the linear activation function, the deep learning neural network will not overfit to small sample size and the use of any number of layers was allowed for such DNN to be trained without the concern of overfitting to the data. The performance of this model in calibration, cross-validation and prediction sets for prediction of TVB-N were presented in Table 2. The result showed that the LDNN model exhibited acceptable performance for the prediction of TVB-N content (R^2^p = 0.853; RSMEP = 3.159 and RDP = 3.001). Moreover, in order to better compare the performance of LDNN algorithm, two well-known models including PLSR and LS-SVM were also established. Although, all of the chemometric models exhibited good performance in the prediction of the TVB-N value (0.82 < R^2^_p_ < 0.9 and 2.5 < RDP < 3). In the calibration set, the PLSR and LS-SVM models showed better performance in comparison with LDNN algorithm (lower RMSC and higher R^2^_C_). However, in the prediction set, the lowest RMSEP and difference between RMSEP and RMSEC as well as the highest RDP obtained in LDNN model (Table 2). This can be considered as a reason for more stability of the LDNN model to predict TVB-N values of rainbow trout fillets. Therefore, Deep learning as a state-of-the-art method for processing large and complicated datasets, showed a promising performance to resolve regression problems and evaluation of TVB-N value of fish fillets. In this regard, Yu et al., (2017) used HSI combined with a deep learning algorithm (stacked auto-encoders (SAEs)) followed by logistic regression (LR) to classify the fresh and stale shrimp based on TVB-N value during cold storage and reported the results showed that the established SAEs-LR model is satisfactory for discriminating freshness grade of the shrimp (R^2^_P_ = 0.858 and RMSEP = 0.19 and RPD = 2.64)^19^. Yu, Wang, Wen, Yang, and Zhang (2019) also correlated the hyperspectral data (900–1700 nm) for determining total volatile basic nitrogen (TVB-N) content in shrimp. They compared Successive projections algorithm (SPA) and deep-learning-based stacked auto-encoders (SAEs) algorithm to select spectral features. The SAEs-LS-SVM and SPA-LS-SVM showed a suitable performance with RPD values of 3.58 and 3.11 respectively which compared with our findings. Deep learning method can learn representational features from the dataset during the training process, and show stronger ability than traditional methods in the current study (RDP > 3).

**Table 2:** Calibration, cross-validation and prediction results of the TVB-N values of rainbow-trout samples by hyperspectral imaging system.

Model	n	Calibration		Cross-validation		Prediction		
		R^2^_C(adj)_	RSMEC	R^2^_CV(adj)_	RSMECV	R^2^_P(adj)_	RSMEP	RDP
LDNN	9	0.849	3.359	0.855	3.116	0.853	3.159	3.001
LS-SVM	9	0.879	3.300	0.870	3.421	0.861	3.530	2.686
PLSR	9	0.891	3.256	0.877	3.061	0.858	3.596	2.636

*R*^*2*^_*C(adj)*_ adjusted determination coefficient of calibration, *R*^*2*^_*CV (adj)*_ adjusted determination coefficient of cross-validation, *R*^*2*^_*P(adj)*_ adjusted determination coefficient of prediction, *RMSEC* root-mean-square errors estimated by calibration, *RMSECV* root-mean-square errors estimated by cross-validation, *RMSEP* root-mean-square errors estimated by prediction, *RDP* residual predictive deviation.

**Figure 3:**
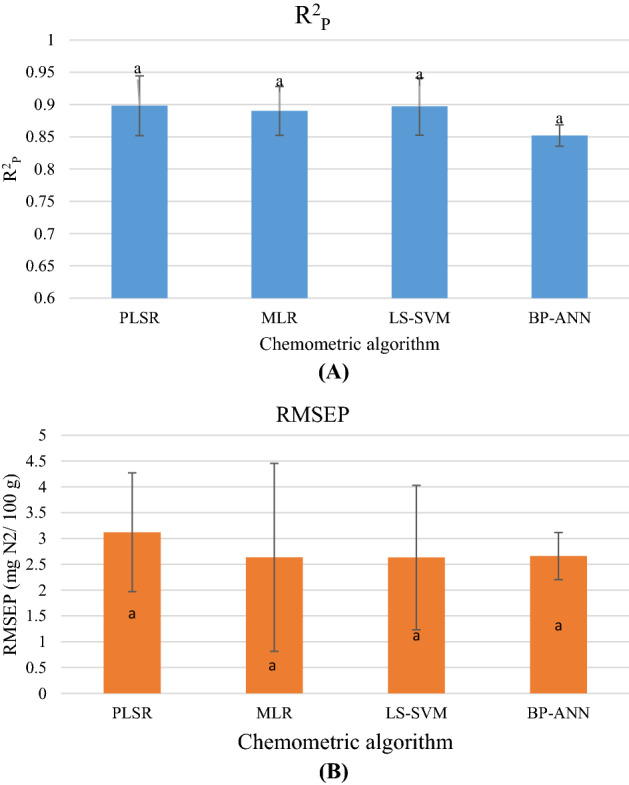
The effect of various chemometric models on **(A)** R^2^_P_; **(B)** RMSEP; Similar small letters indicate non-significant difference between models (*p* > 0.05).

Based on Table 3 and Fig. 3, the performance of LDNN model was comparable with the results of previous works established for prediction of TVB-N value of various meat and seafood products based on hyperspectral imaging systems^5–7,33^.

**Table 3:** Prediction results of the TVB-N values of several meat products by hyperspectral imaging system.

Meat source	Optimal wavebands (n, method)	Results	Algorithm	N of samples	Ref
Grass carp (fish)	420; 466; 523; 552; 595; 615; 717; 850; 955 (9: SPA)	R^2^_p_: 0.91^a^ → 0.89 RMESEP: 2.75 → 2.81	LS-SVM	120	^7^
	420; 466; 523; 552; 595; 615; 717; 850; 955 (9: SPA)	R^2^_p_: 0.81^a^ → 0.90 RMESEP: 5.93 → 2.78	PLSR	120	
Grass carp (fish)	432; 550; 660; 820; 965 (5: SPA)	R^2^_p_:–^a^ → 0.931 RMESEP:— → 1.065	LS-SVM	280	^33^
	432; 550; 660; 820; 965 (5: SPA)	R^2^_p_:–^a^ → 0.921 RMESEP:— → 1.115	MLR	280	
	435; 565; 660; 815; 870; 970 (6: GA)	R^2^_p_:–^a^ → 0.922 RMESEP:— → 1.115	LS-SVM	280	
	435; 565; 660; 815; 870; 970 (6: GA)	R^2^_p_:–^a^ → 0.925 RMESEP:— → 1.098	MLR	280	
Grass carp (fish)	416; 442; 445; 515; 560; 601; 660; 690; 730; 780; 850; 971 (12: GA)	R^2^_p_:–^a^ → 0.917 RMESEP:— → 2.348	PLSR	140	^6^
	416; 442; 445; 515; 560; 601; 660; 690; 730; 780; 850; 971 (12: GA)	R^2^_p_:–^a^ → 0.923 RMESEP:— → 2.280	LS-SVM	140	
	428; 550; 601; 655; 775; 986 (6: PN-GA)	R^2^_p_:–^a^ → 0.956 RMESEP:— → 1.737	PLSR	140	
	428; 550; 601; 655; 775; 986 (6: PN-GA)	R^2^_p_:–^a^ → 0.947 RMESEP:— → 1.846	LS-SVM	140	
Rainbow-trout (fish)	488, 542, 576, 602, 626, 706, 764, 857, 951 (9: GA)	R^2^_p_:–^a^ → 0.857 RMESEP:— → 3.58	PLSR	210	^5^
	488, 542, 576, 602, 626, 706, 764, 857, 951 (9: GA)	R^2^_p_:–^a^ → 0.855 RMESEP:— → 3.59	MLR	210	
	488, 542, 576, 602, 626, 706, 764, 857, 951 (9: GA)	R^2^_p_:–^a^ → 0.862 RMESEP:— → 3.54	LS-SVM	210	
	488, 542, 576, 602, 626, 706, 764, 857, 951 (9: GA)	R^2^_p_:–^a^ → 0.853 RMESEP:— → 3.64	BP-ANN	210	
Rainbow-trout (fish)	481, 524, 554, 595, 629, 696, 721, 768, 896, 958 (10: GA-SR)	R^2^_p_: 0.886 ^a^ → 0.900 RMESEP: 3.086 → 3.006	PLSR	210	^32^
	481, 524, 554, 595, 629, 696, 721, 768, 896, 958 (10: GA-SR)	R^2^_p_: 0.881 ^a^ → 0.894 RMESEP: 3.114 → 3.12	LS-SVM	210	
Cured meat (pork)	-	R^2^_p_: 0.81^a^ → - RMESEP: 5.93 → -	LS-SVM	210	^34^
	553; 583; 643; 675; 709; 749; 908; 937 (9: RC)	R^2^_p_: 0.85 ^a^ → 0.82 RMESEP: 4.92 → 5.38	PLSR		
	553; 583; 643; 675; 709; 749; 908; 937 (9: RC)	R^2^_p_: –^a^ → 0.86 RMESEP: – → 4.73	MLR		
Pork	432; 445; 574; 587; 636; 683; 713; 867; 886 (9: RC)	R^2^_p_: 0.915 ^a^ → 0.936 RMESEP: 2.51 → 2.93	PLSR	186	^38^
Salted pork meat	405; 425; 444; 472; 563; 578; 592; 632; 725; 756 (10: SPA)	R^2^_p_: 0.887 ^a^ → 0.875 RMESEP: 2.278 → 2.42	ANN	140	^39^
Salted pork meat	438; 440; 441; 560; 589; 591; 690; 838 (8: GA-PLS)	R^2^_p_: 0.887 ^a^ → 0.882 RMESEP: 2.278 → 2.31	ANN	140	
Cooked meat	401, 444, 458, 498, 532, 649, 788, 844 (8: SPA)	R^2^_p_: 0.832 ^a^ → 0.836 RMESEP: 2.70 → 2.71	ANN	140	
Cooked meat	463, 464, 490, 538, 788, 917, 918, 960 (8: GA-PLS)	R^2^_p_: 0.832 ^a^ → 0.853 RMESEP: 2.70 → 2.43	ANN	140	
Salted + cooked meats	407, 422, 673, 813, 860, 896, 938, 960, 990 (9: SPA)	R^2^_p_: 0.824 ^a^ → 0.831 RMESEP: 2.84 → 2.70	ANN	280	
Salted + cooked meats	425, 426, 428, 657, 807, 959, 960, 988 (8: GA-PLS)	R^2^_p_: 0.824 ^a^ → 0.854 RMESEP: 2.84 → 2.44	ANN	280	

*SR* stepwise regression, *PN* Physarum network (PN), *GA* genetic algorithm (GA), *RC* regression coefficient, *SPA* successive projection algorithm, *R*^*2*^_*P*_ adjusted determination coefficient of prediction, *RMSEP* root-mean-square errors estimated by prediction, *MLR* multi-linear regression, *PLSR* partial least squares regression, *LS-SVM* least squares support vector machine, *BP-ANN* back-propagation artificial neural network, *ANN* artificial neural network.

^a^The results of quantifying model based on full spectral range.

Figure 3a,b showed the effect of various chemometric algorithms on the predictive power of hyperspectral imaging system. The results of meta-analysis indicated that although linear models averagely showed a higher R^2^_P_ value, the lowest RMSEP was obtained for non-linear model (R^2^_P(linear model)_ = 0.895 ± 0.0417 vs R^2^_P(non-linear model)_: 0.876 ± 0.0406; RMSEP_(linear model)_ = 2.945 ± 1.36 vs. RMSEP_(linear model)_ = 2.648 ± 1.032; P > 0.05). The result was in agreement with the LDNN finding. The results of the meta-analysis did not show any significant difference between the prediction power of various chemometric models. However, the highest R^2^_P_ and RMSEP value was obtained for PLSR model. Since the LS-SVM model showed a relative high R^2^_P_ beside a lower RMSEP value, this model can be considered as more stable than the PLSR one to estimate TVB-N content on fish and meat products. It should be noted that the performance of a chemometric model is a function of several factors: the number of samples and variables; the optimal waveband selection method; the type of samples and chemical structure of them; the hyperspectral imaging wavelength range and etc. In this regard, Cheng et al. (2016) reported that when the SPA method was used for optimal wavelength selection, the best method was obtained for the LS-SVM model, while the GA method was provided the best performance for the MLR model^33^. However, Khoshnoudi-Nia and Moosavi-Nasab et al. (2019) ranked the performance of the various chemometric models to predict TVB-N content of rainbow trout based on R^2^_P_, RMSEP and RDP as follow: LS-SVM > PLSR > MLR > BP-ANN^5^.

**Figure 4:**
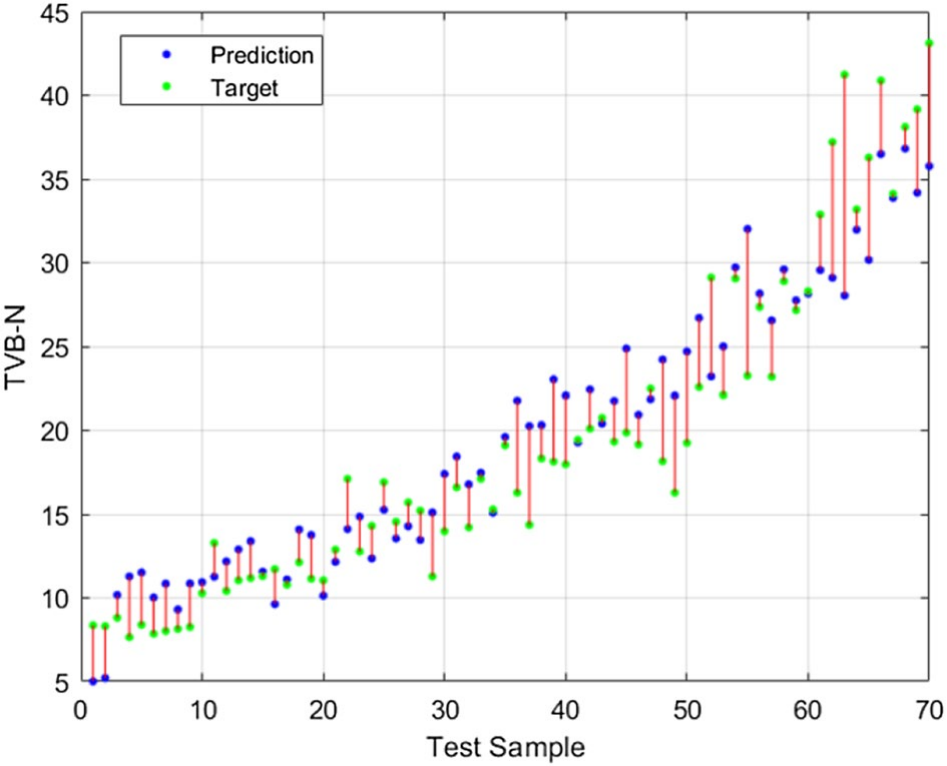
Regression results of LDNN for 70 samples of prediction set.

The difference between RMSEP and RMSEC of LDNN model showed that this model provided a stable and reliable model for prediction of TVB-N value (RMSEP-RMSEC = 0.2 mg N/100 g). Khoshnoudi-Nia and Moosavi-Nasab et al. (2019) also introduced the BP-ANN model (RMSEP-RMSEC = 0.326 mg N/100 g) as a suitable model to evaluate rainbow fish fillet freshness. Moreover, as shown in Fig. 4 LDNN model subjected to some under-fitting and overfitting in the lower and higher value of TVB-N. However, for moderate values (10–20 mg N/100 g) LDNN model showed great potential for TVB-N prediction.

## Materials and methods

### Fish fillets preparation

Forty fresh rainbow-trout (*Oncorhynchus mykiss*) fish (0.75–1.2 kg) were caught in a local aquaculture pond (Bajgah, Shiraz, Fars, Iran) in December 2016 and immediately transported to the Seafood-Processing Research Group laboratory (Shiraz, Iran). Total transport time was 15 min. After rigor mortis, the rainbow trout fishes were filleted and washed with cold water. Fish fillets were cut into a small size and consequently, 210 subsamples were obtained (8.0 × 4.0 × 1.0 cm). All the subsamples were labeled, packaged by and stored at 4 ± 2 ^◦^C for 12 days. To exhibit the suitability of the model, 70% of subsamples were classified in training (calibration) group (140 subsamples) and 30% was used to test the model (prediction set: 70 subsamples)^1^.

### Acquisition and calibration hyperspectral images

The region of 430–1010 nm was considered for analysis. The main components of the HSI system consisted of a mobile platform (stepper motor), hyperspectral imaging unit (Hyper Spectral Imaging (1000) spectrograph, Opt Co., Kashan, Iran), an illumination unit (two tungsten (100 W) light sources and two daylight fluorescent (36 W) lamps at two sides of the stepper motor). The speed of the stepper motor was adjusted on 0.6 mms^-1^ and exposure time of 25 ms, controlled by a computer system (LabVIEW 2011, National Instruments CO. Austin, USA)^13,32^.

Subsamples were placed on the stepper motor to scan by a line-scanning hyperspectral imaging system. The obtained images corrected by black and white reference images to reduce the effect of illumination and “dark” current of the hyperspectral imaging units^2,13,28^.

### Determination of TVB-N content

Immediately after hyperspectral image collection, the samples were analyzed for TVB-N value by a micro Kjeldahl distillation unit (Kjeltec PDU-500, PECO food Co., Iran) as explained by Goulas & Kontominas (2005) using the following equation:^40^.

1 $$ {\text{TVB-N }}\left( {{\text{mg}}/{1}00{\text{ g fish muscle}}} \right) = \frac{{\left( {V_{1} - V_{2} } \right) \times c \times 14}}{{{\raise0.7ex\hbox{${\left( {m \times 5} \right)}$} \!\mathord{\left/ {\vphantom {{\left( {m \times 5} \right)} {100}}}\right.\kern-\nulldelimiterspace} \!\lower0.7ex\hbox{${100}$}}}} \times 100 $$

V_1_ is the titration volume for the tested sample (mL); V_2_ is the titration volume of blank sample (mL); c is the actual concentration of HCl (mol L^−1^); m is the weight of minced muscle (g)^12,40^.

### ROI identification and extraction of spectral data

The region of interests (ROIs) of hyperspectral imaging were identified manually by the software of hyperspectral imaging system (LabVIEW 2011, National Instruments CO. Austin, USA). A mean spectrum for each fish fillet was obtained and used as input data for evaluation TVB-N values of the samples based on a trained deep learning algorithm. Savitzky–Golay (S–G) algorithm was used to decline the noises of extracted average spectrum (by: Unscrambler 10.4; CAMO, Trondheim, Norway)^5^.

### Optimal wavelength selection

Data extracted from hyperspectral images of each fish fillet sample comprises hundreds of contiguous wavebands. However, most of these wavelengths are poorly correlated with TVB-N content. Successive projections algorithm (SPA), as a forward selection method, was used to select the most informative wavelengths. This algorithm starts with one waveband and incorporates a new one at each replication until a specified number of wavebands with minimum redundancy is obtained^41^. The procedure of SPA was conducted in Matlab 2016a software (The MathWorks Inc., Mass, USA).

### Deep regressor model for TVB-N prediction

Our objective is to predict the TVB-N value of fish fillets based on deep learning method. The main approaches for regression analysis include analytical methods and neural network (NN) based methods. The former assumes a mathematical equation and aims at finding the optimum parameters for this equation describing the relationship between variables, while the latter train a NN as a *black box* with the input predictor and the output outcome variables, to estimate their relationship.

#### Artificial neural network (ANN)

**Figure 5:**
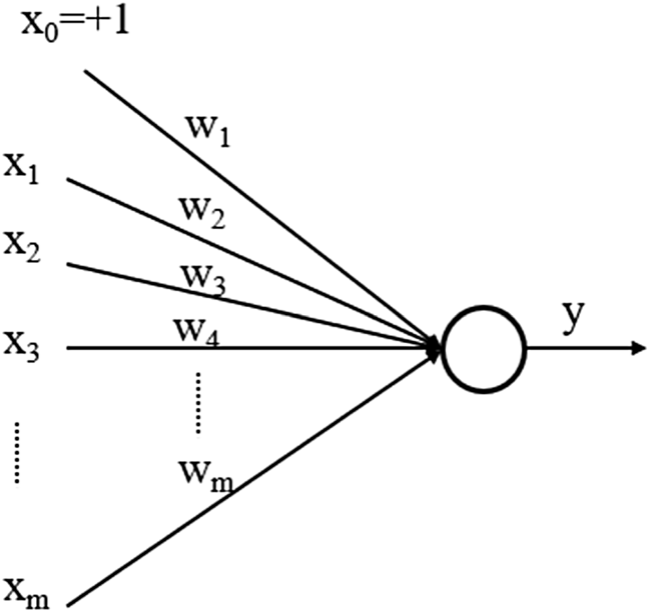
Diagram of an artificial neuron as building block of a ANN. For a given artificial neuron, let there be *m* + *1* inputs with signals *x*_*0*_ through *x*_*m*_ and weights *w*_*o*_ through w_m_. The output *y* of the neuron acts like an input to the neurons in the next layer^42^.

An ANN is based on artificial neurons (i.e., a collection of connected units) which showed in Fig. 5.

Each connection can transmit a signal to other neurons. The output *y* will be computed as^42^:

2 $$y= \varphi (\sum_{j=1}^{m}{w}_{j}{x}_{j})$$

In Fig. 5, the activation functions $\varphi (.)$ determine the type of the function which ANN approximates. If all activation functions are selected to be linear, then we will have a linear ANN (LANN), while by choosing nonlinear activation functions in ANN the ANN provides a nonlinear function approximator. Moreover, if the number of network layers proceeds from some threshold (e.g., 5) we call the NN a Deep Neural Network (DNN).

In this research, DNNs was used for regression of the TVB-N content of fish fillet by a set of existing data. The advantage of using NNs over analytical methods include their efficient inference step and their power in handling the noise in the data.

The training (learning) phase of NNs is performed considering examples, and without being programmed with task-specific rules.

#### Network structure

**Figure 6:**
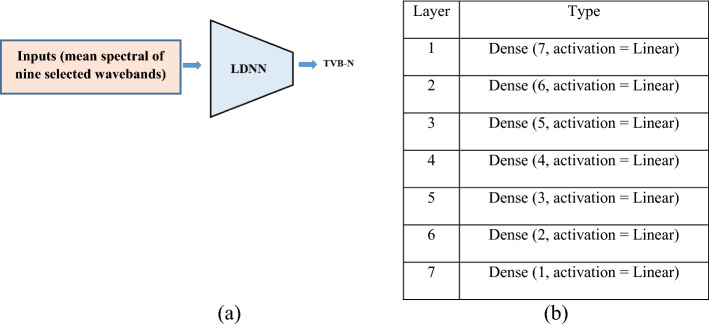
Network structure for first LDNN used in the experiments for regression of TVB-N feature. **(a) **Schematic diagram, **(b)** detailed structure.

Because of the small sample size, training nonlinear NN models for our problem encounters overfitting to the training data. Therefore, we are restricted to linear regression, to find a line (or a more complex linear function) that most closely fits the data according to a specific mathematical criterion (e.g., MSE). The linear models do not overfit to the data because of their simple structure. A seven layers linear Deep Neural Network (MLP) was designed for regression of TVB-N content of fish fillets with the structure was presented in Fig. 6. LDNN model was designed using Keras library and trained it using 140 samples, as the training data and test it using 70 samples as the test data.

### PLSR and LS-SVM models

The calibration between the spectral and TVB-N value was established by PLSR and LS-SVM models to compare the results of them with LDNN performance. The linear models were conducted in Unscrambler 10.4 × software (CAMO, Trondheim, Norway) and LS-SVM model was established in MATLAB R2016a (The Mathworks Inc., Natick, MA, USA).

### Model evaluation

The spectral data selected by the SPA algorithm were considered as input for training (calibration set) and testing (prediction set) the deep learning models. The assessment factors include the adjusted determination coefficient (R^2^_C(adj)_, R^2^_CV(adj)_, and R^2^_P(adj)_), the root mean square error of them (RMSEC, RMSECV and RMSEP) and residual predictive deviation (RDP)^43^.

Generally, a suitable prediction model can be introduced based on the following principles: (i) high value of determination coefficient (poor model: R^2^ < 0.82; good model: 0.82 $\le$ R^2^ $\le$ 0.9; and excellent model: R^2^ > 0.9); (ii) high RPD value (RPD < 1.5: very poor model; 1.5 < RPD < 2.0: poor model; 2 < RPD < 2.5: fair model; 2.5 < RPD < 2.5: good model; RPD > 3: very good model and R > 5: excellent model) (iii) low RMSEs values and (iv) a small difference between RMSEC and RMSEP value^1,44,45^. The building, validation, and evaluation processes of LDNN model were carried out in MATLAB R2016a (The Mathworks Inc., Natick, MA, USA)^5,32^.

### Statistical analysis

TVB-N analysis was conducted in 30 replicate and the data were reported as mean ± standard division (SD). Statistical analyses were carried out by one-way ANOVA. Tukey’s test was applied to determine the significant differences between the means (P ≤ 0.05). Statistical calculations were performed in Minitab Version 17 statistical software (Minitab Inc. Pennsylvania, USA).

### Meta-analysis

Meta-analysis was done based on published studies reporting the evaluation of TVB-N content of meat products based on HSI system (400–1000 nm). The articles with experimental study design (original research), searching on meat and seafood, HSI system (400–1000 nm), TVB-N content (based on regression) and English language were considered as eligible article to review. On the other hands the letters, conference abstracts, patients and review articles as well as the researches classified the meat samples based on TVB-N content were excluded. Thus, eight articles (26 case studies: some articles had compared the two or more algorithms) were selected. A comprehensive literature search was carried out between 20 December 2019 to 20 January 2020 on google scholar databases. The following search terms were applied as keywords and Boolean operator: “hyperspectral imaging” + meat, TVB-N + “hyperspectral imaging”. The search was limited to English language articles. The meta-analysis was carried out based on descriptive analysis (one-way ANalysis Of VAriance (ANOVA)) and comparative analysis (frequency data). Statistical analysis was performed using the Minitab 17.1.0 (Minitab Inc. PA, USA)^46^. The further details were provided in supplementary materials (Supplementary Fig S1 and Supplementary Table S1).

### Statement

All experiments and methods were performed in accordance the approved guidelines of the Shiraz University. All experimental protocols were approved by the Ethics Committee of Shiraz University of Medical Sciences and all experiments were conducted in accordance with the approved guidelines of Iran Veterinary Organization.

The methods were carried out in accordance with the approved guidelines of the University of Sydney Ethics Committee. All experimental protocols were approved by the University of Sydney Ethics Committee.

### Ethical approval

This article does not contain any studies with human participants performed by any of the authors.

## Conclusions

In several previous works, the potential of various deep learning algorithms to classify of seafood and meat products in two freshness grades (fresh and stale) was investigated. The results demonstrated that the hyperspectral imaging system coupled with the deep learning method had great potential for classifying of freshness quality of meat products with a total classification accuracy of more than 90%. However, sometimes the evaluation of freshness index based on numerical output and in a regression, framework is helpful for better decision making and management. As a result, the present study is the first time that a deep learning algorithm was applied to predict a freshness indicator in regression framework. In the calibration set, the PLSR and LS-SVM models showed better performance than the LDNN algorithm. However, in the prediction set, the findings of this study demonstrate that the combination of linear deep learning neural network and the hyperspectral system gave reasonable accuracy for the prediction of TVB-N content in fish fillets (R^2^_P_ = 0.853; RMSEP = 3.159 and RDP = 3.001). Based on the meta-analysis, the results of established prediction system were comparable with the hyperspectral imaging system based on the traditional chemometric analysis. In order to make the DLNN model with higher accuracy and ability, a large amount of data to train the system is necessary. it is the most important challenge for evaluating food product quality based on a deep-learning algorithm and experimental data. Therefore, there is still much work to be done and the results obtained by the SPA-LDNN method would encourage more research efforts on using deep learning as a novel chemometric method for evaluating the freshness quality of food products. Establishing a comprehensive database for a certain fish freshness index, the extraction of modeling features (e.g., optimal wavebands) based on a deep-learning method and the use of other deep-learning algorithms and compare their performance, are some suggested solutions to enhance the accuracy of a hyperspectral system coupled with deep learning algorithms.

## Supplementary Information

Supplementary Information.

## Data Availability

The datasets generated and/or analyzed during the current study are available from the corresponding author on reasonable request.
